# CLINICAL CASE “I finally woke up, it seems like a miracle”

**DOI:** 10.1192/j.eurpsy.2022.556

**Published:** 2022-09-01

**Authors:** H.J. Min

**Affiliations:** CAULE, Psychiatry, León, Spain

**Keywords:** MYELOID ANGIOPATIA, SUBARACHNOID HEMORRHAGE, DEPRESSIVE DISORDER SECONDARY, MODERATE COGNITIVE IMPAIRMENT

## Abstract

**Introduction:**

Clinical case about a 62 year old patient, diagnosed with mixed anxiety-depressive disorder. In current treatment with Paroxetine, Clorazepate and Trazodone. She presents low spirits secondary to a neurological process (stroke + amyloid angiopathy+ epilepsy+ cognitive impairment) and difficulties in performing daily activities. Her daughter reports that she is totally dependent, she spends the whole day in bed. On examination we observe a dull mood, without emotional reactivity. Lack of motivation. Psychomotor slowness.Significant cognitive impairment with difficulties in performing daily activities. Mnesic faults.

**Objectives:**

Clinical case shows radiological and cognitive improvement with vortioxetine

**Methods:**

Last diagnoses: August 2020: HSA OF THE FRONTAL AND PARIETAL GROOVES RIGHTS September 2020:SYMPTOMATIC FOCAL EPILEPSY SECONDARY TO LEFT FRONTOPARIETAL INTRAPARENCHIMATOSUS HEMATOMA December 2020:MYELOID ANGIOPATIA February 2021:SUBARACHNOID HEMORRHAGE March 2021:DEPRESSIVE DISORDER SECONDARY TO MEDICAL ILLNESS - MODERATE COGNITIVE IMPAIRMENT

**Results:**

Spectacular improvement in mood and cognitive deficit. The family reports that after 5 days they noticed the change. They find her more lively, she has returned to doing housework and is autonomous for day-to-day life. She has regained her memory and performed calculation exercises on a daily. The patient says that she has returned to being her usual self, “before I felt like a mummy and now I have finally woken up, it seems like a miracle”.

**Conclusions:**

It has improved much with change of antidepressant, from paroxetine to vortioxetine in patient who show in cranial MRI: New-onset lesion in the right frontal lobe attributable and subarachnoid hemorrhage located in convex furrows ,radiological findings in favor of amyloid angiopathy

**Disclosure:**

No significant relationships.

